# High-Throughput Kinetic Screening of UV and Visible Light-Induced Copper-RDRP in Continuous Flow Using Inline Benchtop NMR Analysis

**DOI:** 10.1021/jacsau.6c00015

**Published:** 2026-04-02

**Authors:** Mia D. Hall, Bo Zhang, Evelina Liarou, Tanja Junkers, David Haddleton

**Affiliations:** † Department of Chemistry, 2707 University of Warwick, Coventry CV4 7AL, U.K.; ‡ School of Chemistry, 2541 Monash University, Clayton, Victoria 3800, Australia

**Keywords:** polymer, copper, RDRP, reaction monitoring, NMR, photopolymerization

## Abstract

Photoinduced copper-mediated reversible deactivation radical polymerization (photoinduced Cu-RDRP) is investigated through a simple automated continuous flow platform enabling rapid kinetic screening via inline NMR measurements. The effects of different molecular weight targets, monomers with various side chain functionalities, changes in solvent, and the ability to form polymer blocks were monitored in real-time. Offline samples were extracted to confirm the validity of the inline analysis (±4%) as well as size-exclusion chromatography (SEC) to show well-controlled polyacrylates (1.09 ≤ *D̵* ≤ 1.28) with good end-group fidelity as demonstrated by matrix-assisted laser desorption/ionization time-of-flight (MALDI-ToF). Low energy visible light could be applied to polymerize polyacrylates using catalytic amounts of appropriate commercially available dyes giving full monomer conversion in only 20 min, signifying the efficiency of a user-friendly platform to optimize and monitor polymer synthesis under mild conditions.

## Graphical abstract

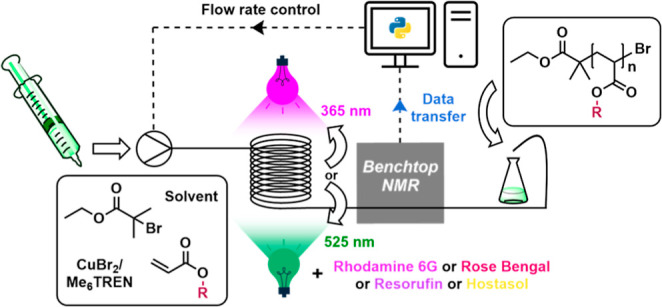

## Introduction

Photochemistry carried out in continuous flow is a powerful approach with potential increases in efficiency and sustainability over traditional batch methods. Photochemical reactions in batch can suffer from limited light penetration, which is exacerbated at higher volumes, and therefore the reaction solution at the vessel wall progresses at accelerated rates compared to the center, resulting in irreproducible results, potential loss of control and inability to scale-up.^1–3^ With a high surface-to-volume ratio, continuous flow facilitates improved mixing, heat dissipation, and safer processing.^4–6^ The use of light enables mild conditions since it is environmentally benign and readily available, allowing for spatiotemporal control and selectivity.^7,8^

Photoinduced reversible deactivation radical polymerizations (RDRPs) have been investigated in continuous flow, in particular photoinduced reversible-fragmentation chain-transfer (RAFT)^6,9–12^ and atom transfer radical polymerization (ATRP).^13–18^ Photoinduced RDRP permits the synthesis of well-defined polymers with pre-established chain lengths, potential for complex architectures and tolerance to many functional groups under relatively mild conditions.^19–26^ Many studies on photoinduced copper-mediated RDRP (Cu-RDRP), also referred to as photo-ATRP, employ ultraviolet (UV) irradiation (λ ∼ 365 nm) due to the absorption of the copper complex in the UV region.^27–30^ Dual-catalytic systems have been developed allowing polymerization under visible and infrared light using a traditional photoinduced Cu-RDRP copper complex alongside a photocatalytic (PC) species, often an organic dye.^31–38^ These dyes are also frequently reported to permit polymerization in the presence of oxygen while enabling deeper light penetration and milder conditions for biomolecular and other sensitive systems.^39,40^ Interestingly, to the best of our knowledge, visible-light-induced dual catalytic PC/Cu-RDRP has not been investigated in flow before, which would enable facile synthesis of polymers efficiently in short timeframes under low energy, mild conditions with the ability to adeptly scale-up if desired.

Junkers and colleagues previously utilized photoinduced Cu-RDRP in continuous flow to synthesize poly­(methyl acrylate) (PMA) under UV irradiation, investigating various chain lengths, chain extensions, and comparison of milli-flow and microflow devices.^15^ High monomer conversion (≈90%) was achieved within only 20 min of residence (reaction) time, producing well-defined polymers (*D̵* ≤ 1.1) with high end-group fidelity and a linear increase in the number-average molecular weight (*M*_n_) as well as the facility to form block copolymers. This study was expanded to explore various monomers and the ability to upscale a reactor from 2 to 16 mL to easily produce over 200 g of well-defined, low dispersity polymer in a relatively short time frame, underpinning the excellent scalability of photoinduced Cu-RDRP.^17^ Haddleton and colleagues investigated photoinduced Cu-RDRP in flow without the need of deoxygenation. By using relatively high amounts of the copper complex, they were able to generate quite precise polymers with a higher initiator efficiency compared to batch reactions.^18^

One key advantage of a continuous flow system is the facile integration of inline and online monitoring instruments, as well as the potential for automation.^41–46^ Real-time monitoring can be implemented for batch reactions and has previously for photoinduced Cu-RDRP;^47^ however, the additional benefits of continuous flow are therefore unavailable, and the execution is more difficult. In situ analysis is particularly valuable as it eliminates human error in sample collection and preparation,^41^ particularly for photochemical reactions where delays could affect samples through unintended light exposure. Inline monitoring specifically allows for ease of analysis without interrupting the reaction flow and a range of inline devices can be used to monitor polymer synthesis such as nuclear magnetic resonance (NMR),^48–50^ Fourier transform infrared spectroscopy (FTIR),^51,52^ mass spectrometry (MS), and UV–vis spectroscopy.^53,54^ NMR is particularly useful, as it provides detailed structural information without the need for calibration between samples. Real-time NMR monitoring can be made accessible through low-field benchtop NMR spectrometers, which typically do not require deuterated solvents. While high-field NMR excels in spectral resolution and sensitivity, particularly for complex and dilute samples, a low-field benchtop NMR spectrometer can monitor the conversion of vinyl monomers to polymer through changes in peak integrals.^6,43^ Benchtop spectrometers also benefit from being smaller and less expensive to maintain and operate.

Girkin and colleagues developed a technique to follow kinetics in flow with real-time measurements,^55^ often denoted transient time sweep kinetics.^42^ By using step changes between different flow rates and consequently residence times, the transitional periods can be analyzed rather than singular measurements at steady-state intervals, enabling a high throughput screening method. This has since been adopted in polymer synthesis to monitor changes in monomer conversion and molecular weight, through inline and online instruments such as NMR, FTIR, electrospray ionization (ESI)-MS, and size-exclusion chromatography (SEC).^41,51,53^ An automated, user-friendly platform was built and implemented in the Junkers’ lab to follow transient time sweeps through inline NMR as well as online GPC.^41,42^ Through a software consisting of Python and Labview codes, the required residence times can be inputted, and the program is able to calculate flow rates, run the experiment, and analyze the generated data in real time. This platform has been employed to observe thermal and photoinduced RAFT polymerization.^5,6,41^ An adaptation of this platform has also been developed to follow kinetics through FTIR for RAFT and ring-opening metathesis polymerization in multiple dimensions.^51^ However, to the best of our knowledge, no work has explored inline analysis of photoinduced Cu-RDRP in continuous flow.

Herein, we investigate photoinduced Cu-RDRP in continuous flow, aided by high throughput screening by ^1^H NMR measurements. Through a simple Python script, we have performed machine controlled transient time sweeps with real-time data analysis. The kinetics and control of different chain lengths are explored as well as various hydrophilic, hydrophobic, and amphiphilic monomers. The end-group fidelity was confirmed by matrix-assisted laser desorption/ionization time-of-flight (MALDI-ToF) and in situ chain extension experiments. Chain extension with a second monomer also demonstrated that block copolymers can be easily produced and analyzed. Finally, through the addition of small quantities of PCs, including dyes that have received little scientific interest as PCs, dual-catalytic PC/Cu-RDRP could be studied under low energy visible light in continuous flow.

## Results and Discussion

Initially, we explored the polymerization of methyl acrylate (MA) with a targeted degree of polymerization (DP_n_) = 25 in a continuous flow set up under λ = 365 nm irradiation using the automated system with real-time NMR monitoring, Figure 1. Short (2 min) to longer (20 min) residence times were screened in the same reactor by adjusting from high to low flow rates. In agreement with Junkers and colleagues,^15^ approximately 90% monomer conversion was achieved within 20 min. The reaction was repeated with increased targeted DP_n_ (50 and 100), again resulting in a final monomer conversion of ∼90%, Figure 2. Offline NMR samples were taken and the conversion calculated to compare with the inline data. Small deviations from the real-time data were observed, with an average error of ±4%. Small inconsistencies between inline and offline data have been previously reported,^6^ attributed to imprecise integration, incorrect baseline correction, or apodization.^56^ However, the largest deviation was measured for the first sample for all three experiments (approximately 7%). This deviation could imply an inaccurately established previous steady state; however, the beginning of the time sweep experiments reveals a very rapid reaction rate.

**Figure 1:**
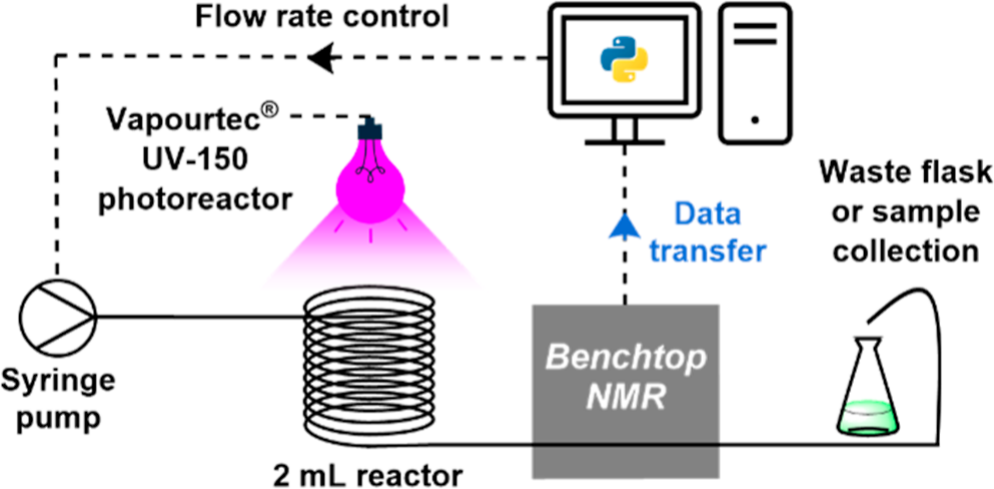
Automated polymerization setup with real-time monitoring, featuring a syringe pump, Vapourtec photoreactor, benchtop 80 MHz NMR, and final waste/sample collection.

**Figure 2:**
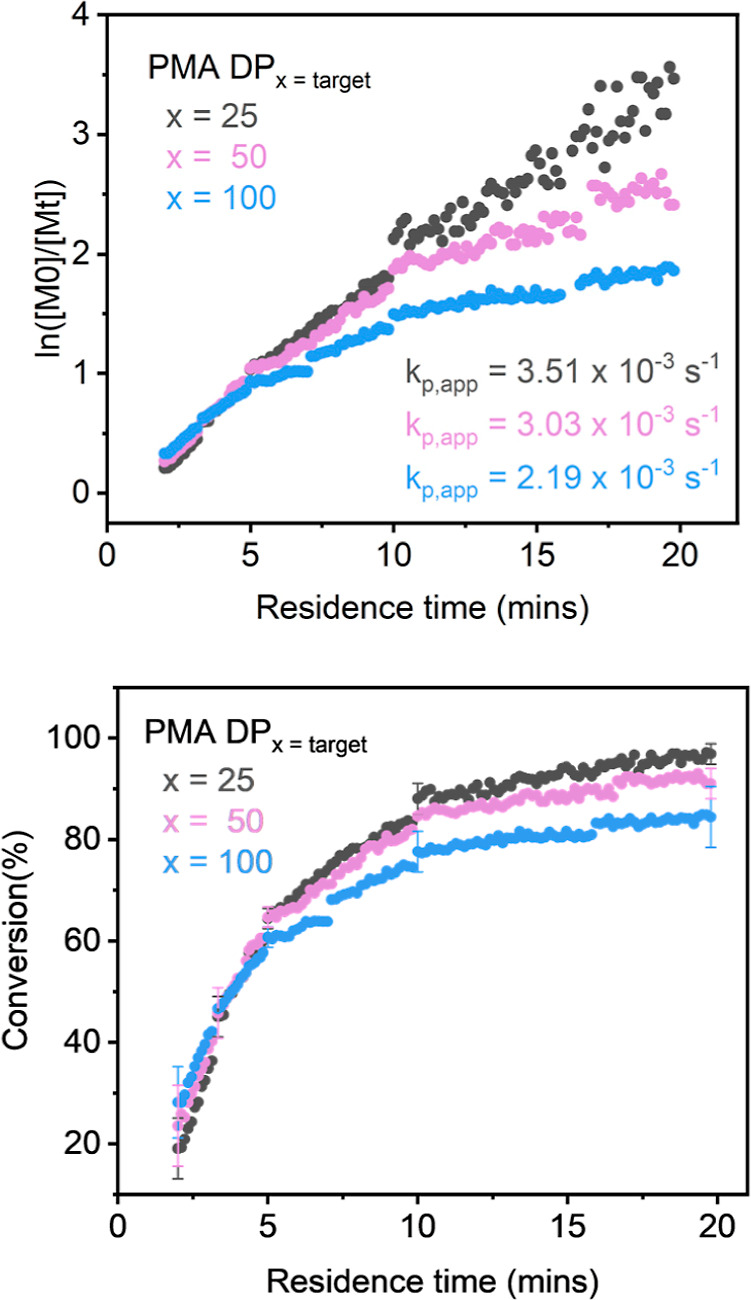
Top: kinetic plot of transient time sweeps of PMA with different degrees of polymerization (DP) targets, synthesized via photoinduced Cu-RDRP with [MA]/[EBiB]/[CuBr_2_]/[Me_6_Tren] = *x*:1:0.02:0.12 in DMSO 50% (v/v) through continuous flow with automated steady-state residence times of 2, 3.3, 5, 10, and 20 min. Bottom: transient time sweep of residence time against conversion for PMA at different DPs, with error bars indicating deviations in inline NMR compared to offline NMR.

Therefore, since the scan rate is not instantaneous, the polymerization proceeds too fast to measure without some deviation. Interestingly, not all three experiments displayed the expected linear first-order kinetics over the full time scales of the experiments, Figure 2(top). While PMA_25_ displayed relatively linear kinetics consistent with a well-controlled photo Cu-RDRP across the whole time sweep, PMA_50_ and PMA_100_ only exhibited similar rates in the first 5 min, followed by curvature. This is indicative of termination and a reduction in the concentration of growing chains. It is noted that previous inline analysis studies have demonstrated that a high throughput, real-time screening can be more efficient for kinetic analysis,^57^ as a much smaller number of data points are collected compared to inline monitoring which could potentially result in nonlinear kinetics appearing linear. Therefore, with our own definition, any distinct curvature of a time sweep where the curve deviates by >15% from the overall linear fit is considered nonlinear. The deviations for PMA_50_ and PMA_100_ are significant, with the overall slope of PMA_50_ differing by 35% compared to the curve while PMA_100_ differs by 45%. Therefore, the apparent polymerization rate coefficient (*k*_p,app_) was determined from the first mostly linear slopes for each DP up to 10 min to provide initial *k*_p,app_ values. The *k*_p,app_ of PMA_25_ in the first 10 min is measured to be 3.51 × 10^–3^ s^–1^ and as expected, the *k*_p,app_ decreased with increasing DP_n_.

Well-defined polymers were observed through the analysis of offline samples, with relatively low dispersity (≤1.11) and narrow molecular weight distributions for all three experiments after 20 min, Figure 3A. A decrease in the dispersity of the polymers over time with an increase in monomer conversion is evident, in line with the expectation of a well-controlled photoinduced Cu-RDRP, Figure 3B. While there was overall good agreement between the calculated and experimental *M*_n_ for all the experiments, a deviation was observed for PMA_100_ at higher conversion. While this discrepancy might often be considered negligible and an accepted deviation from the ideal behavior for a RDRP, this further validates a polymerization with a decreasing radical concentration at higher conversions.

**Figure 3:**
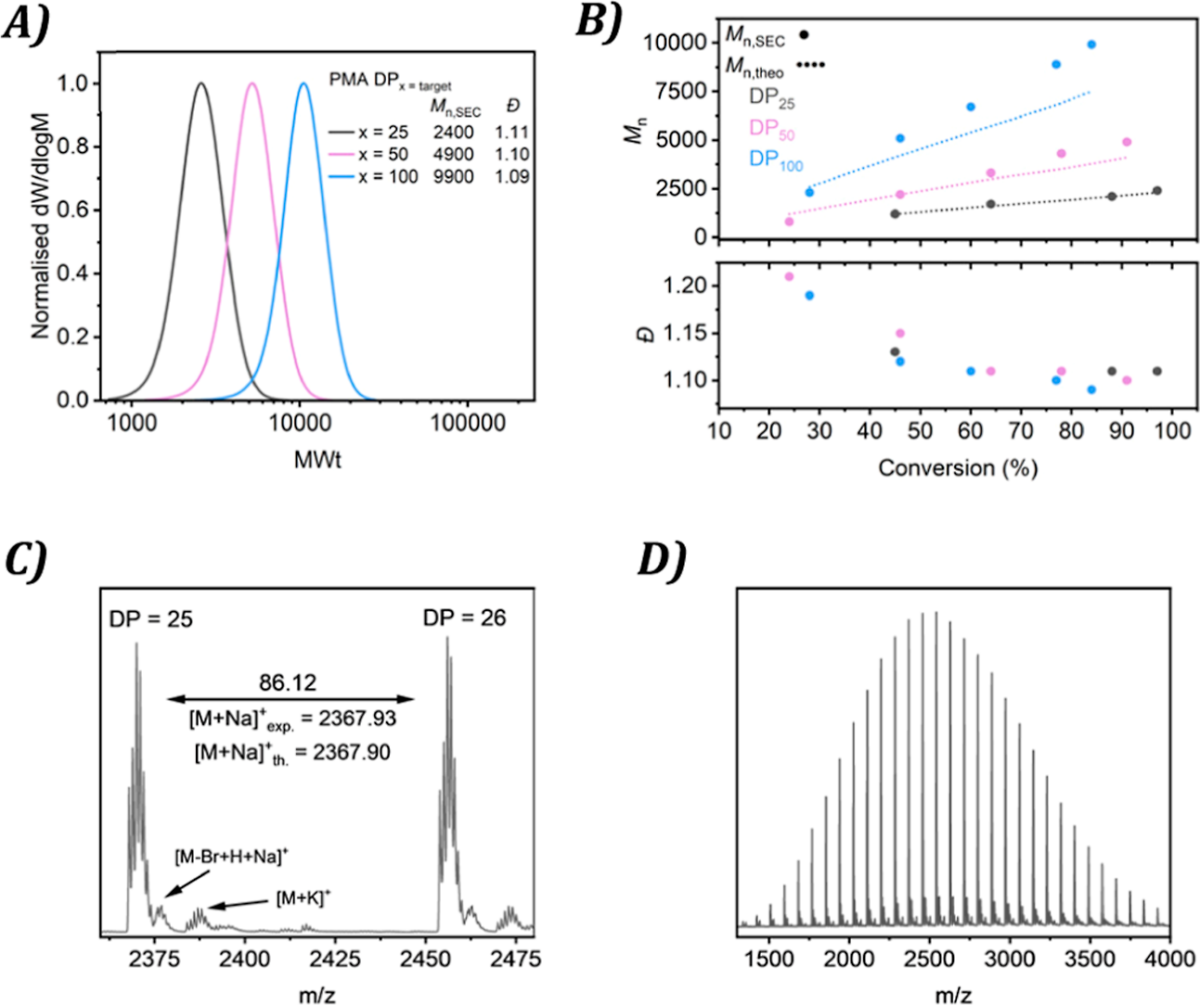
(A) SEC traces of different molecular weights (MWts) of PMA polymerized by photoinduced Cu-RDRP in flow. (B) Theoretical *M*_n_, actual *M*_n_, and dispersity against conversion for the different PMAs polymerized. (C,D) MALD-ToF of PMA_25_.

An essential characteristic of a well-controlled RDRP is the retention of active chain-ends. MALDI-ToF-MS can confirm the presence of a bromine ω-terminus; therefore, it was suitable to analyze PMA with a DP_n_ value of 25. With one predominant distribution separated by the repeating unit of MA and the characteristic isotopic pattern for a bromine terminated polymer, good end-group fidelity was evident, Figure 3C,D. A low abundant distribution of a polymer with a hydrogen end-group was also detected, which might also explain why at higher molecular weights a discrepancy between theoretical and experimental *M*_n_ was observed, despite symmetrical SEC traces demonstrating great control. However, the intensity of the dead chain peak is relatively minimal, approximately only 10% peak height of the bromine-capped chains.

We then expanded the scope of inline monitoring of photoinduced Cu-RDRP to a range of hydrophilic, amphiphilic, and hydrophobic monomers, many of which are of specific interest for their functionality, often allowing for biocompatibility, shape recovery, and antifouling properties, respectively, Scheme 1.^58,59^ Hydrophilic monomers were first explored, Figure 4A and Table 1, including ethylene glycol methyl ether acrylate (EGA), poly­(ethylene glycol) methyl ether acrylate (PEGA_480_), di­(ethylene glycol) methyl ether acrylate (DEGA), and 2-hydroxyethyl acrylate (HEA). Poly­(ethylene glycol) phenyl ether acrylate (PEGPA_324_) was also tested as an amphiphilic monomer. Overall, the monomers showed a linear dependence of ln­([M_0_]/[M_*t*_]) on time, indicating first-order kinetics expected of a controlled radical polymerization. However, the kinetic plots for PEGA_480_ and DEGA showed evidence of early termination, with some curvature in the kinetic plot at longer residence times. Therefore, the *k*_p,app_ for PEGA_480_ and DEGA will be considered for the first 10 min only. EGA displayed a faster reaction rate (*k*_p,app_ = 3.24 × 10^–3^ s^–1^) with high monomer conversion, narrow molecular weight distribution, and good agreement between the experimental and theoretical *M*_n_. HEA exhibited the fastest *k*_p,app_ (4.01 × 10^–3^ s^–1^), however, a significant deviation in the molar mass was observed (*M*_n,SEC_ = 8300 g/mol, *M*_n,theo_ = 3100 g/mol) after 20 min residence time. A low-molecular-weight shoulder was apparent at all residence times, suggesting some fast and early termination possibly due to relatively slow deactivation. The experimental *M*_n_ is approximately double the theoretical *M*_n_ but this is unlikely to be due to termination by combination, as shown by the shift in molecular weight from 5 to 10 min, Figure S6D. This deviation in *M*_n_ could instead be explained by the differences in the hydrodynamic radius of the relatively hydrophilic PHEA to the linear PMMA standards utilized in our DMF GPC, which is in agreement with a previous work.^60^ We also calculated *M*_n_ using ^1^H NMR (2500 g/mol), Figure S7. Gas chromatography (GC)–MS confirmed low levels of impurities in the “as-received” HEA monomer, suggesting that purification was not required, Figure S8. The only discernible disadvantage of employing as-received HEA was a slight increase in dispersity (*D̵* = 1.23) compared to previous research where purified HEA was used.^17,25^ However, control was maintained overall for PHEA with relatively symmetrical SEC traces observed. Polymerization of HEA was also carried out in water as a solvent using the water-soluble initiator 2-hydroxyethyl-2-bromoisobutyrate (HEBiB), however, there was a loss of control, evident by the increased dispersity (*D̵* = 1.57), Figure S6E. PEGPA_324_ is a useful monomer with a long hydrophilic chain capped by a hydrophobic ring, producing polymers with amphiphilic properties. After 20 min, the polymerization of PEGPA_324_ achieved 88% monomer conversion, and despite a higher dispersity (*D̵* = 1.28), the theoretical molecular weight aligned closely with the experiment, suggesting control was preserved.

**Scheme 1:**
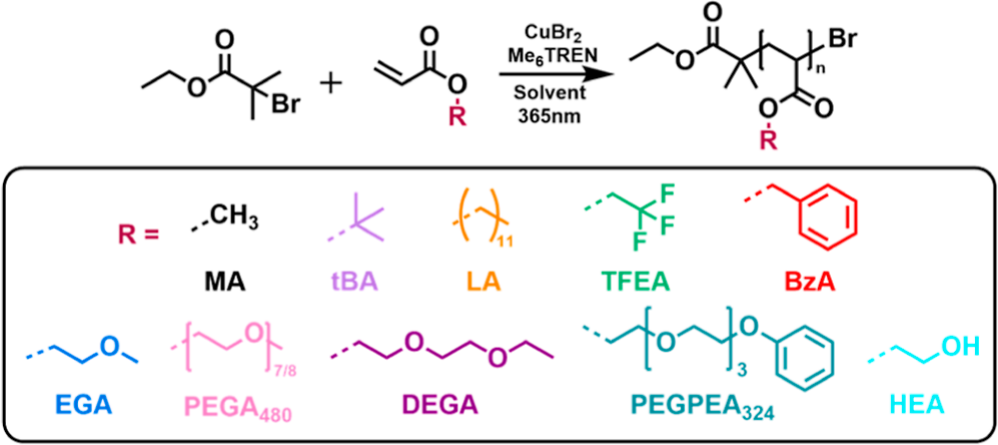
Reaction Scheme of Photo Cu-RDRP and the Different Monomers That Were Explored for Photo-Induced Cu-RDRP

**Figure 4:**
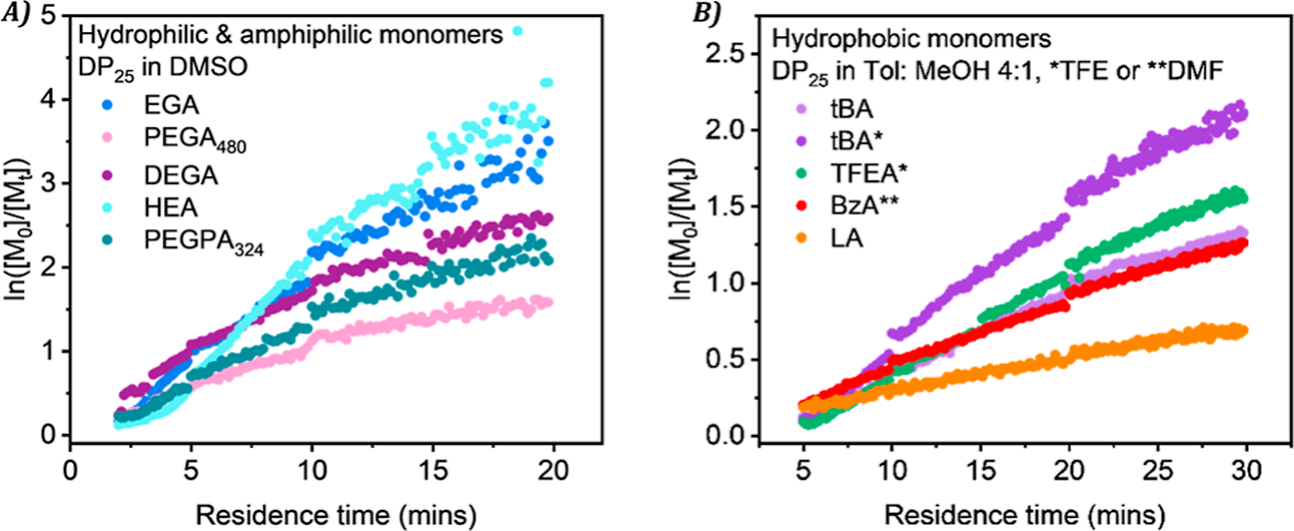
Kinetic plots of the polymerization of (A) hydrophilic and amphiphilic monomers and (B) hydrophobic monomers by photoinduced Cu-RDRP in continuous flow with inline NMR analysis.

**Table 1:** Different Monomers Polymerized Using Photo-Cu-RDRP in Continuous Flow^a^

entry	monomer	solvent	conversion (%)^b^	*M*_n,theo_ ^b^ ^,^ ^c^	*M*_n,exp_ ^b^ ^,^ ^c^	*D̵* ^b^ ^,^ ^c^	*k*_p,app_ (×10^–3^ s^–1^)
1	EGA	DMSO	97	3300	3400	1.14	3.24
2	PEGA_480_	DMSO	80	9800	7600	1.12	1.76^f^
3	DEGA	DMSO	93	4600	3300	1.20	2.74^f^
4^d^	HEA	DMSO	99	3100	8300	1.23	4.01
5^d^ ^,^ ^e^	HEA	H_2_O	87	2700	8100	1.57	1.72
6^d^	PEGPA_324_	DMSO	88	7300	7700	1.28	1.99
7	*t*BA	Tol/MeOH (4:1)	74	2600	2600	1.12	0.86
8	*t*BA	TFE	88	3000	3500	1.12	1.40
9	TFEA	TFE	79	3900	3900	1.09	1.07
10	BzA	DMF	72	2800	2800	1.14	0.71
11	LA	Tol/MeOH (4:1)	50	3300	3300	1.12	0.35

a [M]/[EBiB]/[CuBr_2_]/[Me_6_TREN] = [25]:[1]:[0.02]:[0.12] in 50% (v/v) solvent.

b Data for the polymer at the final residence time.

c Determined from THF SEC analysis unless stated otherwise.

d Determined from DMF SEC analysis.

e HEBiB used instead of EBiB.

f *k*_p,app_ measured for the first 10 min of residence time.

Hydrophobic functional monomers including *tert*-butyl acrylate (*t*BA), trifluoroethyl acrylate (TFEA), benzyl acrylate (BzA), and lauryl acrylate (LA) were then explored in a variety of alternative solvents, Figure 4B and Table 1. Monomers with hydrophobic or semifluorinated moieties are known to form insoluble polymers in DMSO, therefore more suitable solvents were chosen for each monomer. The final residence time for the transient time sweeps was increased up to 30 min as less polar solvents coordinate and solubilize the copper complex less readily. *t*BA was polymerized in two different solvents, toluene/methanol at a 4:1 ratio (Tol/MeOH) and trifluoroethanol (TFE). In Tol/MeOH, the monomer conversion reached 74% in 30 min with *k*_p,app_ = 0.86 × 10^–3^ s^–1^ compared to in TFE where the rate was faster with a *k*_p,app_ of 1.40 × 10^–3^ s^–1^ and 88% monomer conversion, despite displaying the same control (*D̵* = 1.12). For TFEA, a *k*_p,app_ of 1.07 × 10^–3^ s^–1^ and 79% monomer conversion was achieved within 30 min, also exhibiting a low dispersity. BzA and LA both established the lowest *k*_p,app_ values while still obtaining well-defined polymers (*D̵* = 1.14 and 1.12, respectively). Overall, the hydrophobic monomers displayed linear kinetics, while the predicted and experimental molecular weights aligned closely.

As the bromine end-groups were shown to be predominantly maintained on the chain during polymerization by MALDI-ToF, there is an opportunity to form in situ chain extensions and block copolymers via sequential monomer addition. A block of PMA with DP_n_ of 50 was first synthesized in continuous flow under UV irradiation with a residence time of 20 min, before the polymer solution was divided and an aliquot of MA with DMSO added to one stream and EGA with DMSO added to the second. Measuring the integral difference between the vinyl and polymer peak before the chain extension enabled monomer conversion to be calculated smoothly in real-time. For macroinitiator chain extension with MA, first-order kinetics were established with 53% monomer conversion in 20 min, demonstrating retained control and active chains, Figure 5A. The formation of a block copolymer of PMA with EGA was then monitored, again displaying a linear relationship between ln­([M_0_]/[M_*t*_]) and time as well as yielding a well-defined block copolymer in 20 min with 43% monomer conversion and low dispersity (*D̵* = 1.19). The SEC traces confirmed a shift toward higher molecular weight as expected, Figure 5B,C. Comparable theoretical and measured molecular weights at each targeted residence time corroborates the accuracy of the conversion calculation in situ. This verifies inline NMR as an exemplary and accurate real-time monitoring device, as more complicated polymer solutions can easily be analyzed and monitored. The precise tailoring of block copolymers can be performed with tuning of flow rates or other parameters instantaneously, as also shown recently with inline FTIR monitoring.^61^

**Figure 5:**
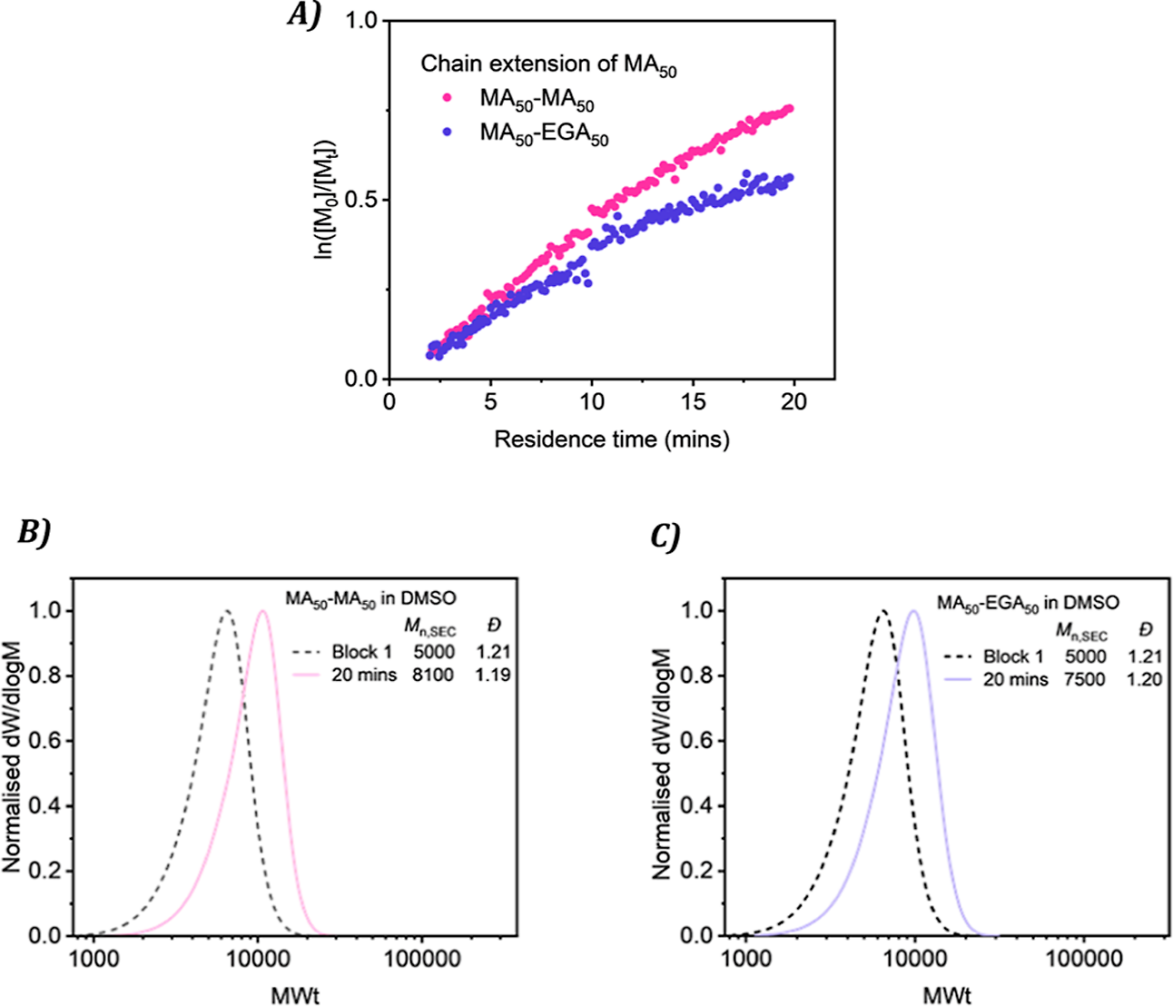
Chain extension experiments with PMA_50_ were conducted in continuous flow through photoinduced Cu-RDRP (A) kinetic plot of chain extension with additional MA (pink) and EGA (purple). (B) SEC traces of PMA and PMA–MA showing a shift in molecular weight. (C) SEC traces of PMA and PMA–EGA showing a shift in molecular weight.

Inspired by the growing interest in visible-light-induced Cu-RDRP,^39^ we conducted polymerizations under green light in continuous flow using inline ^1^H NMR analysis. Four different commercially available dyes were selected as photoredox catalysts: rhodamine 6G (RD 6G), Rose Bengal (RB), Hostasol, and resorufin, Figure 6A. RD 6G and RB were chosen due to their previous success for photoinduced Cu-RDRP under green light, needing only parts per billion quantities to induce polymerization.^34^ Hostasol and resorufin, conversely, have had very little attention as PC dyes. Only recently was Hostasol utilized to perform photoinduced Cu-RDRP under green light as an incorporated dual PC initiator to produce fluorescently labeled well-defined polymers.^38^ Resorufin has not been thoroughly investigated as a photoredox catalyst for photo RDRP, most likely as it is primarily researched as a fluorescent probe. A study on metal-free ATRP using resorufin under green light has recently been published, sparking interest in the phenoxazine dye for photoinduced Cu-RDRP.^62^

**Figure 6:**
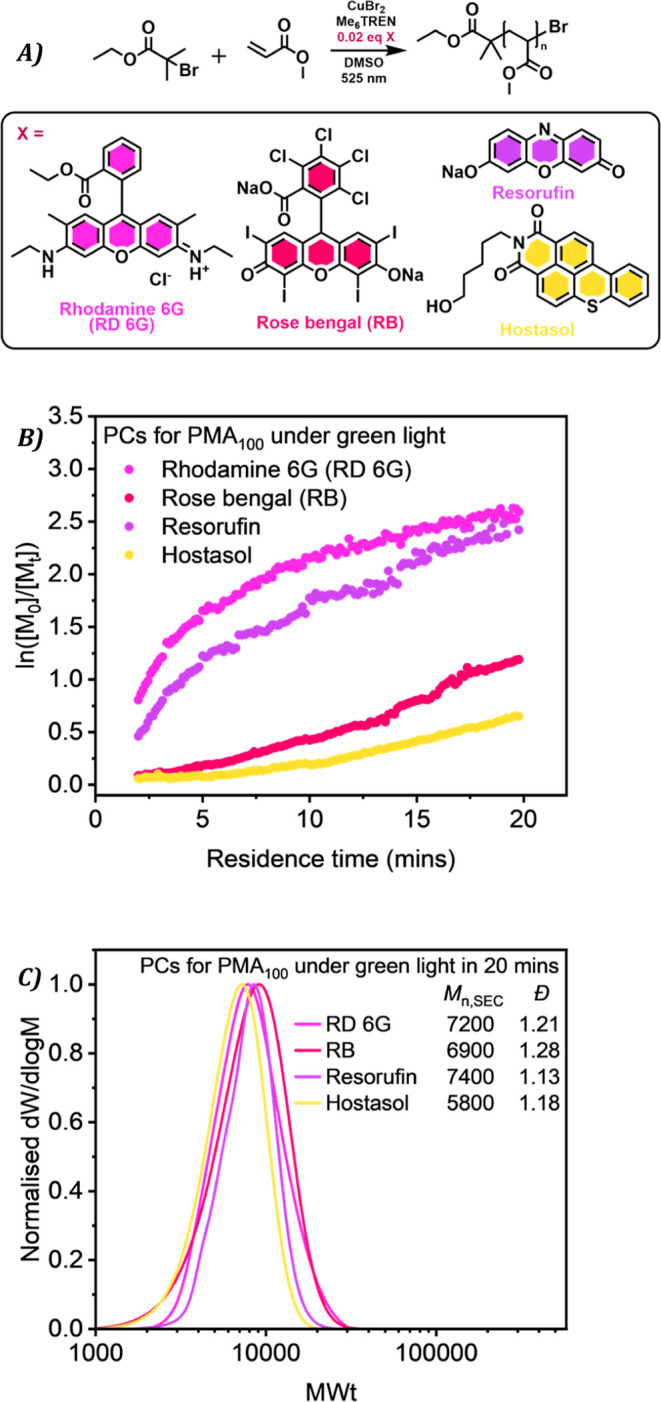
(A) Scheme of polymerization of MA via photoinduced Cu-RDRP in continuous flow with catalytic amounts of different organic dyes under green light irradiation with [MA]/[EBiB]/[CuBr_2_]/[Me_6_Tren]/[PC] = 25:1:0.02:0.12:0.02 in DMSO 50% (v/v). (B) Kinetic plots of transient time sweeps of visible-light-induced Cu-RDRP of MA. (C) SEC traces for different MA polymers synthesized after 20 min residence time.

Thus, catalytic/small amounts (1 mM) of the four dyes were added to mixtures of MA with a DP_n_ = 100 and subjected to irradiation at λ = 525 nm in continuous flow, Figure 6B. Polymerization with RD 6G progressed rapidly at the beginning, then slowed after approximately 10 min, displaying nonlinear kinetics. Despite this, the dispersity was relatively low at 1.21. As previously reported,^34^ RD 6G experiences no induction period suggesting the activator species is rapidly regenerated. Therefore, considering a low deactivation rate in combination with a substantial absorption overlap with a green LED, Figure S11, it is likely that the control was compromised. With the RB dye, the monomer conversion reached 69% after a short induction period, furnishing a higher final dispersity (*D̵* = 1.28) than RD 6G but a better first-order plot than RD 6G. Interestingly, resorufin dye also displayed nonlinear kinetics, with an initial rapid polymerization up until 5 min followed by a constant apparent rate of polymerization, while achieving the lowest dispersity of 1.13 after 20 min. However, a nonfluorescent mixture emerged from the reactor even after only 2 min residence time, suggesting some alteration in the resorufin species (Figure S14). This may be the result of the redox equilibrium where resorufin reduces to dihydroresorufin, a nonfluorescent compound. Therefore, despite diminished fluorescence and color, controlled polymerization continues through the equilibrium. Hostasol proceeded with the lowest observed *k*_p,app_ following an induction period of approximately 5 min but furnished a well-controlled polymer with a narrow molecular weight distribution (*D̵* = 1.18). Hostasol has the most limited absorption overlap with the green LED, Figure S11, thereby explaining the relatively low rate even after 20 min. While all dyes participate in dual-catalytic PC/Cu-RDRP to synthesize polymers with narrow, symmetrical SEC traces under green light in ≤20 min, Figure 6C, the two previously unstudied dyes displayed improved control over the polymerization. This not only affirms the benefits of employing small amounts of inexpensive dyes to polymerize under lower energy wavelengths but broadens the scope of known dyes that can perform efficiently and precisely.

## Conclusions

In summary, we report the real-time analysis of photoinduced Cu-RDRP of acrylates in continuous flow. Transient time-sweep kinetics were streamlined by using a simple, user-friendly platform to input variables and run and analyze the experiments. The large, generated data sets enabled apparent propagation rates to be easily determined. Various molecular weights and monomers were targeted to emphasize the versatility of this analysis, while MALDI-ToF and in situ chain extensions confirmed good end-group fidelity. The use of flow chemistry with online monitoring allows for the collection of real time data, giving the potential to use the information in a machine leaning (ML) feedback loop in the future. This also allows for the collection of data points over very small-time intervals, allowing for the observation of nuances that can be missed when data is collected with longer time periods in between acquisition. Use of the transient time sweep kinetics using step changes between residence times enabling high throughput screening, which again accelerates the data collection, facilitates real time reaction optimization. The advantages of visible light over higher energy radiation could be utilized by the addition of a larger range of inexpensive organic dyes, furnishing well-defined polymers rapidly under lower energy green light in continuous flow.

## Supplementary Material



## Data Availability

Full data sets available at https://wrap.warwick.ac.uk/197260/.

## Notes

Experimental details, data for the different wavelengths, NMR, SEC traces, difference in conversion calculated, comparison intensity, transient time sweeps, kinetic plot, GC-MS trace, different monomers polymerized, and emission spectra (PDF)

## Abbreviations

RDRP: reversible deactivation radical polymerization
RAFT: reversible atom-fragmentation transfer
ATRP: atom transfer radical polymerization
Cu-RDRP: copper-mediated reversible deactivation radical polymerization
UV: ultraviolet
PC: photocatalyst
PMA: poly­(methyl acrylate)
*M*_n_: number average molecular weight
NMR: nuclear magnetic resonance
FTIR: Fourier transform infrared spectroscopy
MS: mass spectroscopy
ESI: electrospray ionization
MALDI-ToF: matrix-assisted laser desorption/ionization time-of-flight
DP_n_: targeted degree of polymerization
*k*_p,app_: apparent polymerization rate coefficient
EGA: ethylene glycol methyl ether acrylate
PEGA_480_: poly­(ethylene glycol) methyl ether acrylate
DEGA: di­(ethylene glycol) methyl ether acrylate
HEA: 2-hydroxyethyl acrylate
PEGPA_324_: poly­(ethylene glycol) phenyl ether acrylate
GC: gas chromatography
EBiB: ethyl 2-bromoisobutyrate
HEBiB: 2-hydroxyethyl 2-bromoisobutyrate
Me_6_TREN: tris­[2-(dimethylamino)­ethyl]­amine
tBa: *tert*-butyl acrylate
TFEA: trifluoroethyl acrylate
BzA: benzyl acrylate
LA: lauryl acrylate
Tol/MeOH: toluene and methanol
TFE: trifluoroethanol
DMSO: dimethyl sulfoxide
DMF: dimethylformamide
RD 6G: rhodamine 6G
RB: Rose Bengal
