# Supplementary material for: High-Throughput Kinetic Screening of UV and Visible Light-Induced Copper-RDRP in Continuous Flow Using Inline Benchtop NMR Analysis
Source: JACS Au. 2026 Apr 2;6(4):2387–95. doi: 10.1021/jacsau.6c00015 (PMC13126163; doi:10.1021/jacsau.6c00015)
Supplement: Supplementary file 1 [file au6c00015_si_001.pdf]

High-throughput kinetic screening of UV and visible light-induced copper-RDRP in continuous flow using inline benchtop NMR analysis

Mia D. Hall,[ab] Bo Zhang,[b] Evelina Liarou,[a] Tanja Junkers,[b]* David Haddleton[a]*

[a] Department of Chemistry, University of Warwick, Coventry CV4 7AL, United Kingdom

[b] School of Chemistry, Monash University, Clayton, VIC 3800, Australia

Supporting info

Materials

Methyl acrylate (MA, 99%), tert-butyl acrylate (t-BA, 98%), ethylene glycol methyl ether acrylate (EGA, 98%), poly(ethylene glycol) methyl ethyl acrylate ($PEGA_{480}$, 97%), di(ethylene glycol) ethyl ether acrylate (DEGA, ≥90%), 2-hydroxyethyl acrylate (HEA, 96%), poly(ethylene glycol) phenyl ether acrylate ($PEGPA_{324}$, 98%), rhodamine 6G (Rd 6G, 99%), rose Bengal (RB, 95%), resorufin sodium salt (Resorufin, ≥98%), ethyl α-bromoisobutyrate (EBiB, 98%), copper(II) bromide (Cu(II)Br2, 99%) and all the solvents were purchased from Sigma-Aldrich and used as received. Lauryl acrylate (LA, 90%) was purchased from Sigma-Aldrich and passed through a plug of basic alumina immediately before use. Benzyl acrylate (BzA, 98%) was purchased from Cornelolens and used as received. 2,2,2-Trifluoroethyl acrylate (TFEA, 99%) was purchased from Apollo Scientific and used as received. "Hostasol" (thioxantheno[2,1,9-dej]isochromene-1,3- dione) was supplied by Clariant and Hostasol alcohol (Host-OH) 2-(8-hydroxy-3,6-dioxaoctyl)thioxantheno[2,1,9-dej]isoqiunoline-1,3-dione was synthesised according to the literature and stored in the dark prior to use.[1] Tris-(2-(dimethylamino)ethyl)amine (Me_6Tren) was synthesized according to the literature and stored at 4°C prior to use.[2]

Instrumentation

^{1}H Nuclear Magnetic Resonance – Low field. ^{1}H inline NMR spectra were recorded on a low field benchtop NMR spectrometer (80 MHz Magritek Spinsolve Ultra) with version 2.3.6. The reaction monitoring suite (RMX) was set with a reaction loop of 17 s consisting of 1 scan, an acquisition time of 6.4s, a repetition time of 15 s and a pulse angle of 90°. Before each experiment, the spectrometer was shimmed using a 41 min "powershim" with a standard solution of 1:9 D_2O: H_2O. All spectra were auto-phased, baseline corrected and integrals picked prior to analysis. Monomer conversions were calculated via ^{1}H NMR spectroscopy by comparing the integrals of monomeric vinyl protons to polymer signals. Integral peaks selected and conversion calculated as previously reported.[3,4]

^{1}H Nuclear Magnetic Resonance- High field. ^{1}H offline NMR spectra were recorded on Bruker DPX-300 or DPX400 spectrometers, using samples dissolved in deuterated chloroform ($CDCl_3$) or deuterated dimethyl sulfoxide (DMSO) obtained from Sigma-Aldrich. Chemical shifts are given as δ in ppm downfield from the internal standard tetramethylsilane (TMS) δ = 0 ppm. Monomer conversions were calculated via ^{1}H NMR spectroscopy by comparing the integrals of monomeric vinyl protons to polymer signals. All spectra were analysed using ACD/NMR processor.

Size exclusion chromatography. THF SEC measurements were carried out on an Agilent Infinity II MDS instrument with differential refractive index (DRI), viscometry (VS), dual angle light scatter (LS) and dual wavelength UV detectors. The system was equipped with 2 x PLgel Mixed C columns (300 x 7.5 mm) and a PLgel 5 µm guard column. The eluent used was THF with 0.01 % butylated hydroxytoluene (BHT) as an additive. Samples were run at 1 mL/min at 30 °C and were filtered through a PTFE membrane with a pore size of 0.2 µm before injection. A conventional calibration was made using narrow molecular weight poly(methyl methacrylate) (11 standards between 2,210,000- 1010 Da) and polystyrene (12 standards between 364,000-160 Da) from Agilent EasiVials. Experimental molar mass ($M_{n,SEC}$) and dispersity ($Đ$) values of synthesized polymers were determined by this calibration using Agilent GPC/SEC software.

DMF SEC measurements were carried out on an Agilent Infinity II MDS instrument with differential refractive index (DRI), viscometry (VS), dual angle light scatter (LS) and dual wavelength UV detectors. The system was equipped with 2 x PLgel Mixed D columns (300 x 7.5 mm) and a PLgel 5 µm guard

column. The eluent used was DMF with 5 mM ammonium fluoroborate (NH_4BF_4) as an additive. Samples were run at 1 mL/min at 50°C and were filtered through a PTFE membrane with a pore size of 0.2 µm before injection. A conventional calibration was made using narrow molecular weight poly(methyl methacrylate) (10 standards between 530,000- 500 Da) from Agilent EasiVials. Experimental molar mass ($M_{n,SEC}$) and dispersity ($Đ$) values of synthesised polymers were determined by this calibration using Agilent GPC/SEC software.

Matrix-assisted laser desorption/ionization time-of-flight. Polymer samples and poly(ethylene glycol) (PEG) calibrants were prepared in THF at a concentration of 10 mg/mL, with 1 mg/mL of NaI as the cationising agent. These samples were then mixed at a ratio of 1:1 with a 40 mg/mL solution of trans-2-[3-(4-tert-butylphenyl)-2-methyl-2-propenylidene] malononitrile (DCTB) in THF. 0.5 µL of each sample was then applied to the MTP 384 ground steel target plate and analysed using either a Bruker Autoflex or Ultraflex II ToF/ToF Analyser, with a nitrogen laser delivering 2 ns laser pulses at 337 nm with positive ion ToF detection using an accelerating voltage of 25 kV.

UV-Vis spectroscopy. UV-Vis spectra were recorded on an Agilent Technologies Cary 60 UV-Vis spectrometer in the range of 200-1100 nm using a quartz cuvette (purchased from Starna) with 10 mm optical length.

Gas chromatography mass spectrometry. GC-MS spectra were recorded on a Shimadzu GC2014 equipped with a Shimadzu AO20i autosampler. The instrument was equipped with a crossbond 5% diphenyl/ 95% dimethyl polysiloxane Thames Restek Rtx-5MS column (length 30 m, 0.25 mm ID, 0.25 µL thickness) with helium as the carrier gas at a column flow rate of 0.72 ml min^{-1}. 1 µL of sample was inject with an injection temperature of 250 °C. The heating profile was 60 to 320 °C with a rate of 10 °C min^{-1} and then held at 3 min. The solvent cut off time was 1 min. Samples were prepared at a concentration of 1 mg ml^{-1} in methanol.

Automated platform and set-up.

A Vapourtec® UV-150 photoreactor was used, connected to a Vapourtec E-Series flow chemistry system. The tubular reactor ($V_{reactor}$ = 2 mL) setup consisting of PFA tubing (1/16'' OD, 1 mm ID) was wrapped around a cartridge before being placed inside the reactor unit, where a Vapourtec LED connected to a provided power supply can be inserted into the top. A cooling system is attached which uses cold air blown over ice to cool the LED, with temperature constantly monitored. Reaction solutions are pumped into the reactor via a Legato 100 syringe pump, using 10 mL Hamilton gas-tight syringes, preflushed with N_2. Tubing was threaded through a benchtop 80 MHz NMR to a waste bottle, which could be manually swapped to collect samples at given intervals. The volume between the end of the photoreactor and the NMR measurement point ($V_{deadvolume}$ = 0.9 mL) was inputted to account for detector delay and this volume of each steady state was collected between sweeps for offline measurements. The flow rates for the time sweeps were pre-programmed via self-written code (Python), and NMR data was collected in real-time with conversion calculated. The script was adapted from recent literature,[5] and is now available here (https://github.com/PRDMonash/Only-NMR-through-only-Python-tranisent-timesweep).

GENERAL PROCEDURE FOR REACTIONS

Typical reaction of photo-induced Cu-RDRP of MA (targeted DP_n=100) in 50% v/v DMSO in flow

A vial was charged with $CuBr_2$ (4.5 mg, 0.02 mmol, 0.02 eq), Me_6TREN (32.4 uL, 0.12 mmol, 0.12 eq) and DMSO (9 mL, 100 eq). MA (9 mL, 0.1 mol, 100 eq) and EBiB (147 uL, 1 mmol, 1 eq) were then added and the vial was sealed with a septum. The mixture was then purged with nitrogen for 15

minutes. DMSO on its own was added to a separate vial and purged also. The only-DMSO was transferred to a 10 mL gas-tight syringe, and the reaction tubing was flushed. Then, the reaction mixture was drawn up into two 10 mL gas-tight syringes, and the reaction was allowed to commence with the automated platform previously described, with the syringe swapped halfway through the experiment to enable constant flow of the mixture. Conversion was measured in real time on the inline benchtop 80 MHz NMR. Offline samples were collected at each steady state between the flow rates and conversion was measured using ^{1}H NMR on the offline NMR and SEC analysis was conducted after the samples having been passed through neutral alumina for the removal of copper salts.

DATA ANALYSIS, NMR AND LED INFORMATION

Table S1: Data for the different wavelengths used in the Vapourtec® UV-150 photoreactor.

Wavelength	Measured irradiance (mW/cm^2)[a,b]
365	6.25
525	6.29

[a] Thorlabs S142C photodiode power sensor measured irradiance at specific wavelengths. [b] Wavelengths were measured through an opening in the reactor wall, with the power sensor positioned facing inwards at this specified point.

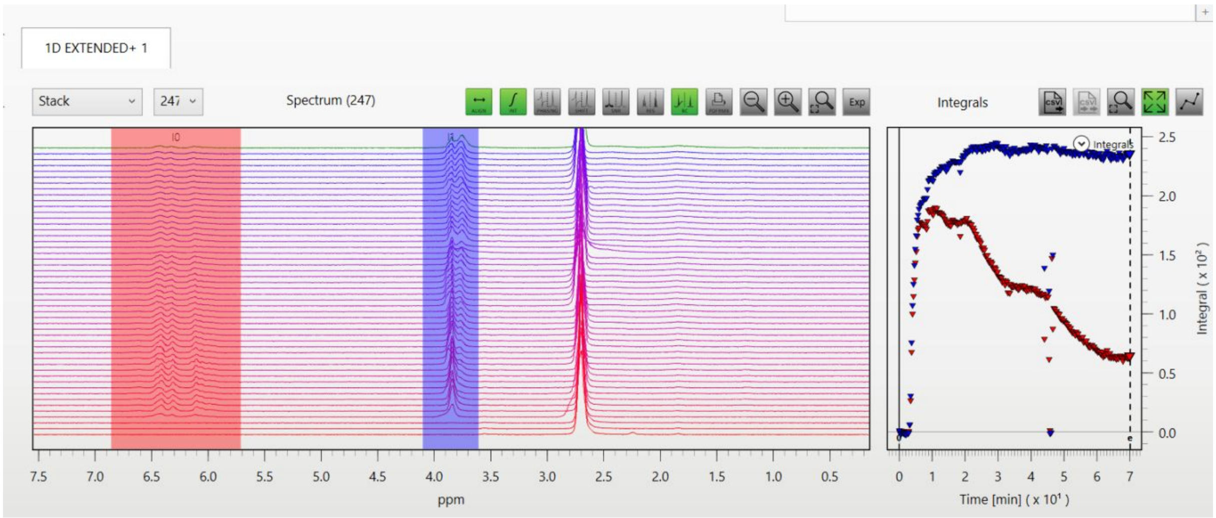

Figure S1: Stacked ^{1}H NMR showing in the change in peak integrals from the first (bottom of the stack) to the last ^{1}H NMR at 20 min for PMA (top of stack) and changes in integrals displayed on the right-hand side. Vinyl peaks were integrated from ~6.8 ppm to 5.7 ppm and polymer peak from ~4.1 ppm to 3.6 ppm.

$$1.\quad Conversion\ (\%) = 100 \times \left(1 - \frac{\int 1H_{vinyl}}{\int 1H_{polymer}}\right)$$

Equation S1: Monomer conversion calculation where 1H vinyl represents the vinyl monomer integral at any given time and 1H polymer for the polymer peak.

$$2. \quad t_{res} = \frac{V_{reactor}}{f_1} + t_m\left(1 - \frac{f_2}{f_1}\right)$$

Equation S2: Calculation of how reaction times (t_m) are converted into residence time (t_{res}) as described previously,[6] where $V_{reactor}$ is the volume of the reactor, f_1 is the previous flowrate while f_2 is the current flowrate. The next spectra are assigned by adding the time per spectra, 17 seconds, to t_m.

$$3. \quad t_{m,0} = t_{m,change} + (f_2 \times V_{dead})$$

Equation S3: Calculation for the start of the timesweep ($t_{m,0}$) as described previously,[6] where the amount of time to pass the dead volume (V_{dead}) is added to the time the flow rate is changed.

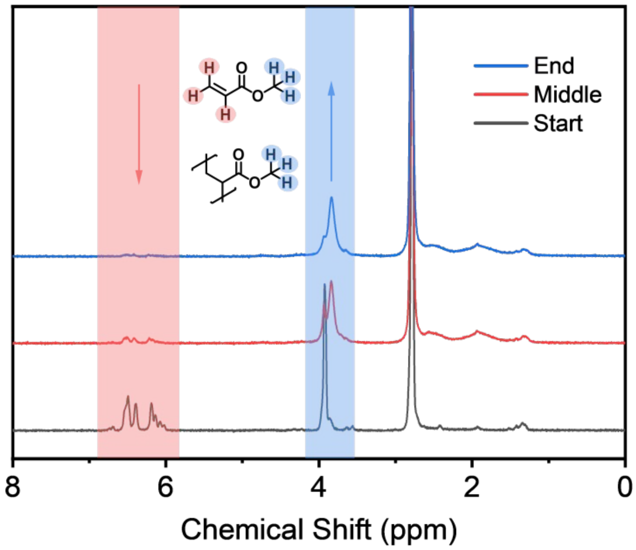

Figure S2: Examples of 80 MHz ^{1}H NMRs of PMA throughout an experiment, with NMRs chosen at the beginning of the experiment/ early in the experiment (short residence time), middle of the experiment and towards the end of the experiment (long residence time). Highlighted areas show connection to either vinyl peaks or monomer and polymer peaks.

Supplementary figures

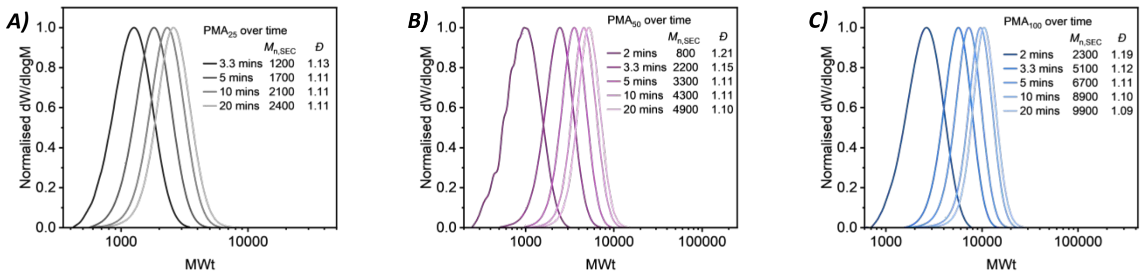

Figure S3: SEC traces showing the evolution of MWt of PMA at targeted degrees of polymerisation (DP_n) = A) 25, B) 50 and C) 100 by photo Cu-RDRP under continuous flow.

Table S2: Difference in conversion calculated through inline benchtop 80 MHz ^{1}H NMR compared to high field 400 MHz for different targeted degrees of polymerisation at different residence times.

DP_n	Time (mins)	Conversion (%) 80 MHz	Conversion (%) 400 MHz	Deviation (%)
25	2	19	13	6
	3.3	45	49	4
	5	64	62	2
	10	88	85	3
	20	97	95	2
50	2	23	15	8
	3.3	46	40	6
	5	65	62	3
	10	85	85	0
	20	91	94	3
100	2	28	21	7
	3.3	47	46	1
	5	61	62	1
	10	78	82	5
	20	84	88	4

Table S3: Comparison intensity of Br- and H- terminated polymers from the MALDI-ToF

DP	Peak intensity	Relative amount of H to Br
20	322663	11.1%

	35817	
23	514115<br>53030	10.3%
26	601287<br>56555	9.4%
29	553477<br>53216	9.6%
32	438195<br>48342	11.0%

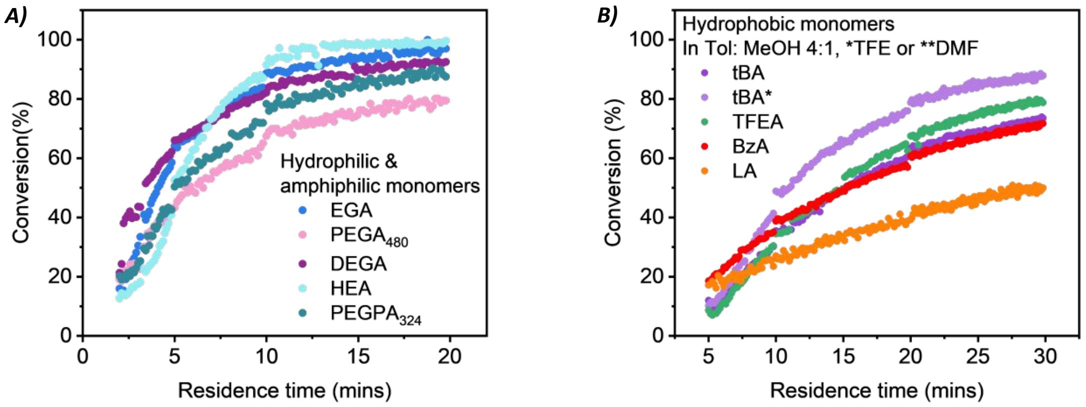

Figure S4: Transient timesweeps showing residence time (mins) against conversion (%) by photo-induced Cu-RDRP of A) hydrophilic and amphiphilic monomers and B) hydrophobic monomers in continuous flow by inline NMR analysis.

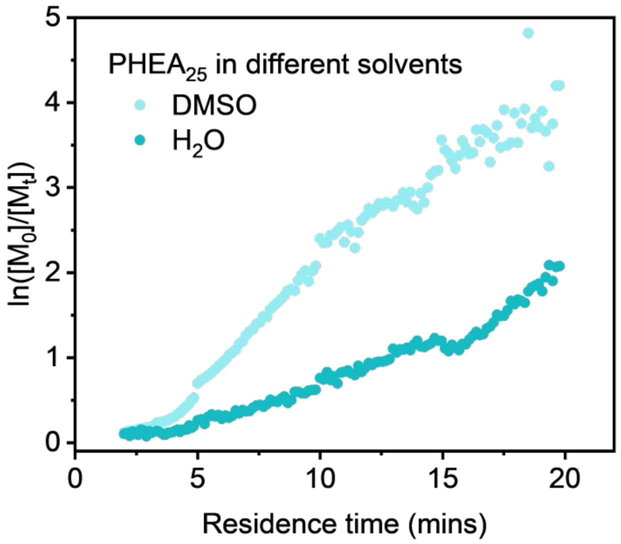

Figure S5: Kinetic plot of photo-induced Cu-RDRP OF HEA with DP_n = 25 in DMSO and water.

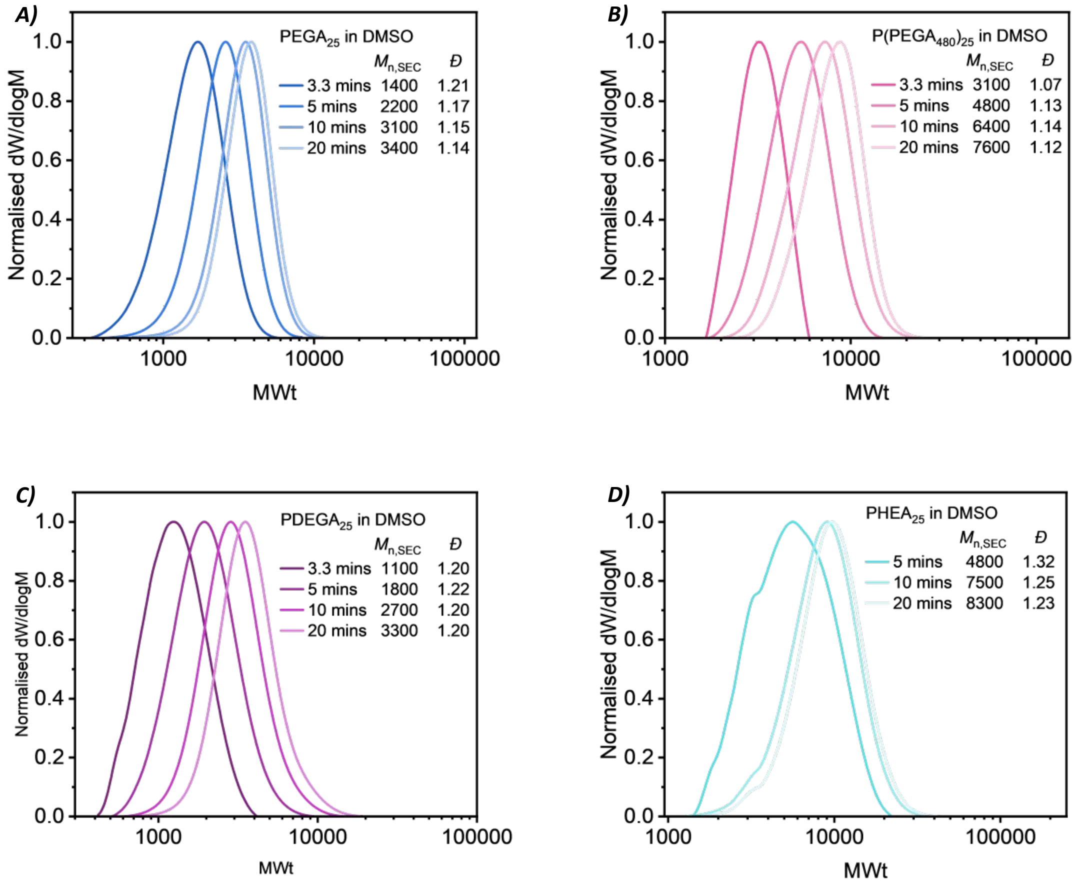

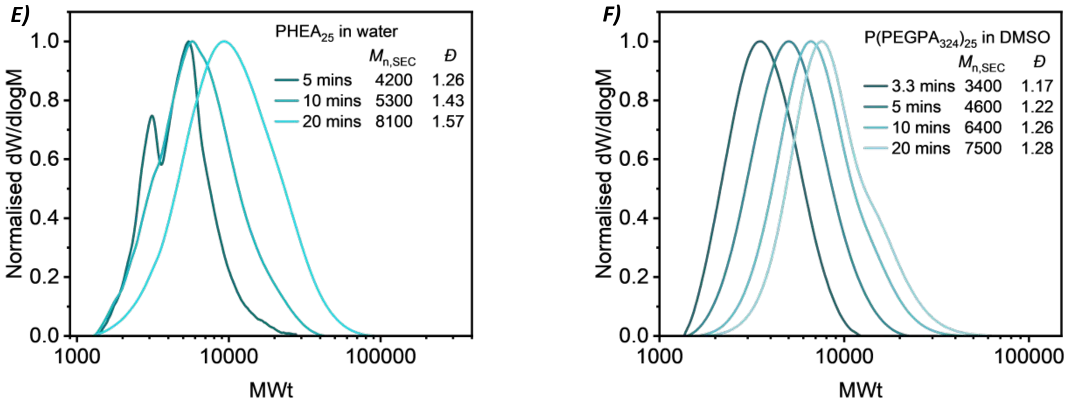

Figure S6: SEC traces showing the evolution of MWt of A) $P(EGA)_{25}$ B) $P(PEGA_{480})_{25}$ C) $P(DEGA)_{25}$ D) $P(HEA)_{25}$ in DMSO E) $PHEA_{25}$ in water and F) $P(PEGPA_{324})_{25}$ synthesised by photo-induced Cu-RDRP in continuous flow.

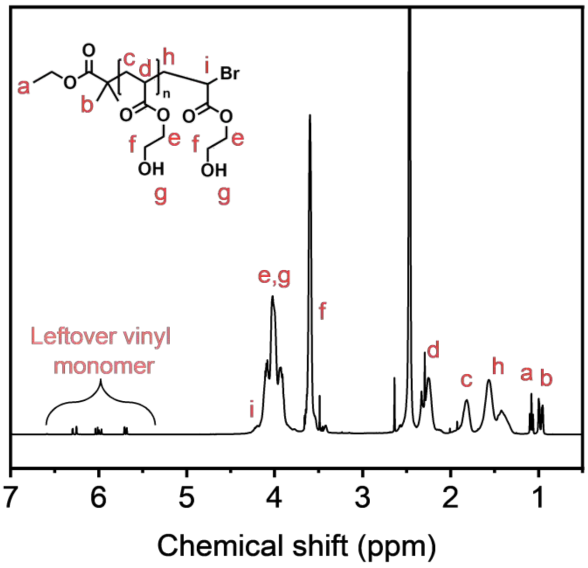

Figure S7: 400 MHz ^{1}H NMR of $PHEA_{25}$ after 20-minute residence time synthesised by photo-induced Cu-RDRP in continuous flow, used to calculate M_n.

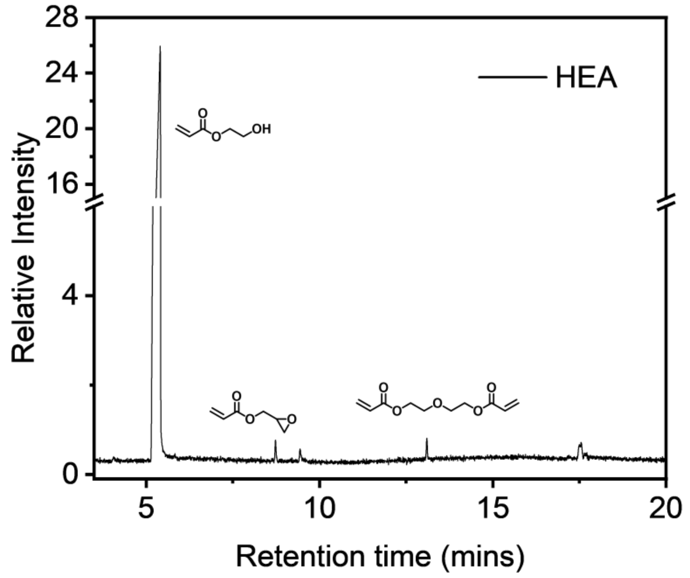

Figure S8: GC-MS trace of HEA with suggested impurity peaks.

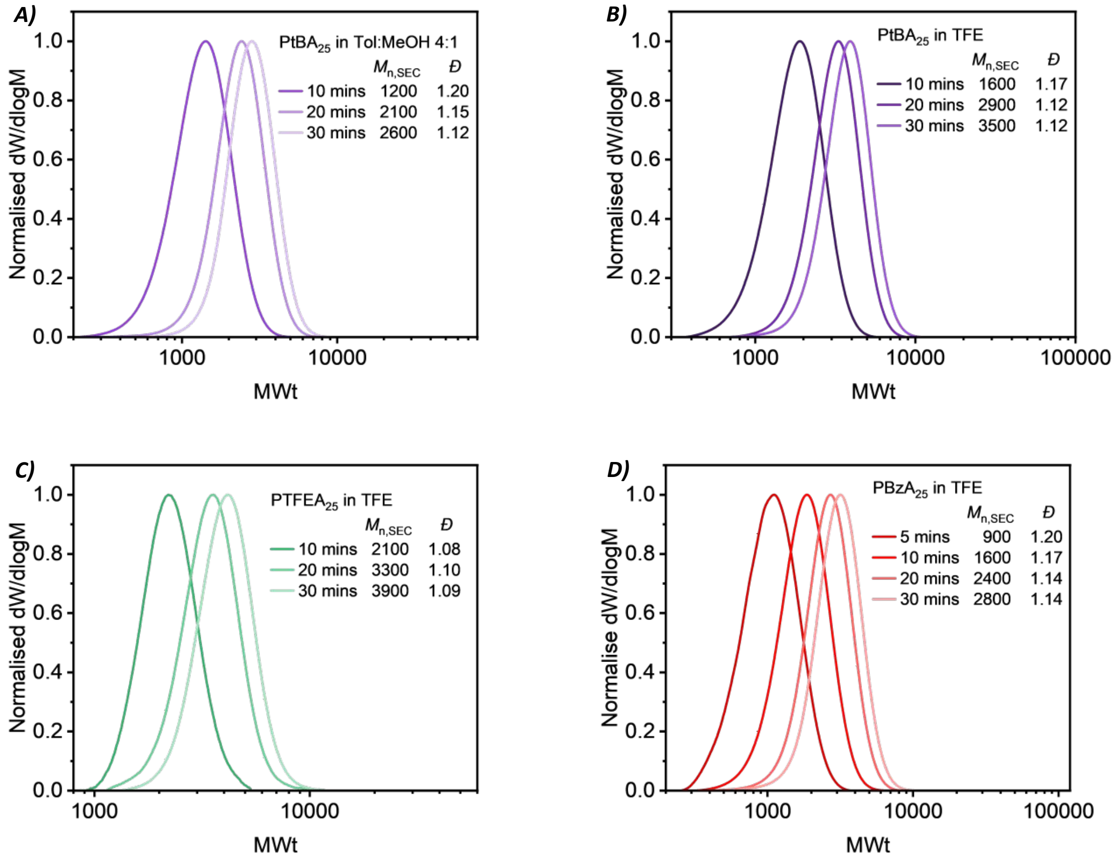

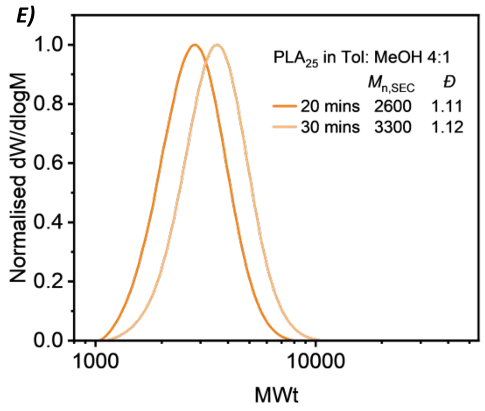

Figure S9: SEC traces showing the evolution of MWt of A) $P(tBA)_{25}$ in Tol:MeOH 4:1 B) $P(tBA)_{25}$ in TFE C) $P(TFEA)_{25}$ D) $P(BzA)_{25}$ and E) $P(LA)_{25}$ synthesised by photo-induced Cu-RDRP in continuous flow.

Table S4: Different monomers polymerised using a PMA_{50} macroinitiator through photo Cu-RDRP in continuous flow

Polymer blocks	Conversion (%)[a]	$M_{n,theo}$[a, b]	$M_{n,SEC}$[a,b]	$Đ$ [a,b]	$k_{p,app}$ ($\times 10^{-3}$ s^{-1})
PMA-MA	53	7300	8100	1.19	0.67
PMA-EGA	43	7800	7500	1.20	0.48

[a] Data for the final polymer at 20-minute residence time. [b] Determined from THF SEC analysis.

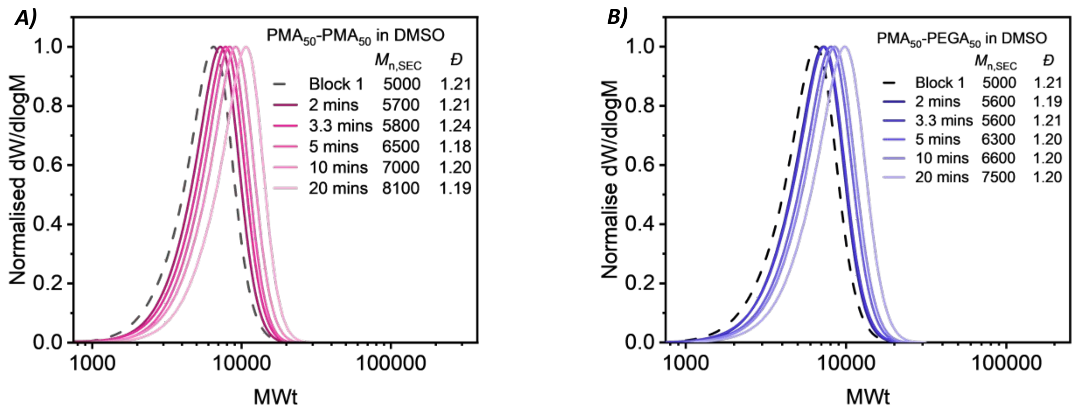

Figure S10: SEC traces showing the evolution of MWt through chain extension of PMA_{50} with A) MA and B) EGA synthesised by photo-induced Cu-RDRP in continuous flow.

Table S5: Green light induced Cu-RDRP of PMA_{100} using small quantities of different photocatalytic organic dyes.

PC	ppm	Conversion (%)[a]	$M_{n,theo}$[a,b]	$M_{n,SEC}$[a,b]	$Đ$[a,b]
RD 6G	525	93	8200	7200	1.21
RB	1100	69	6100	6900	1.28
Resorufin	233	92	8100	7400	1.13
Hostasol	427	48	4300	5800	1.18

[a] Data for the final polymer at 20-minute residence time. [b] Determined from THF SEC analysis.

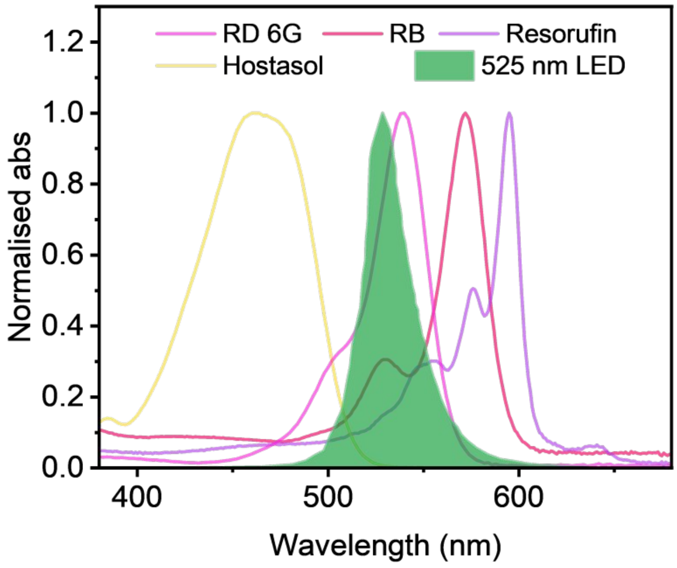

Figure S11: Emission spectra of photocatalytic dyes compared to the normalised distribution of the green LED.

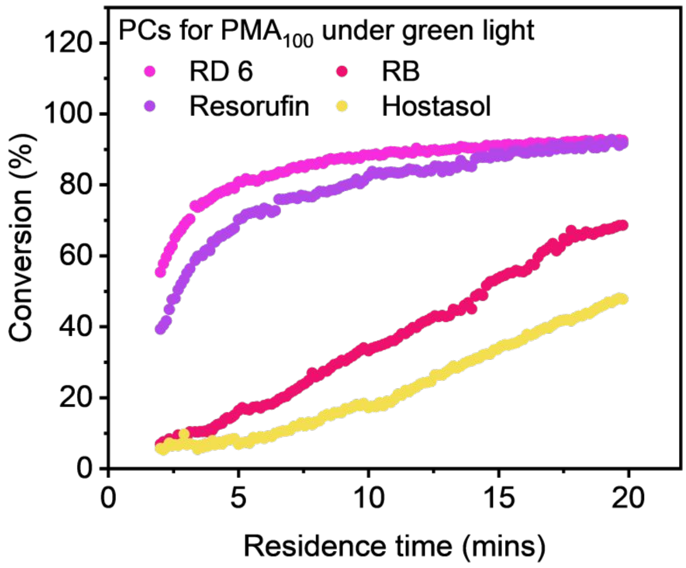

Figure S12: Transient timesweeps showing residence time (mins) against conversion (%) by green light-induced Cu-RDRP of MA using small quantities of photocatalytic organic dyes.

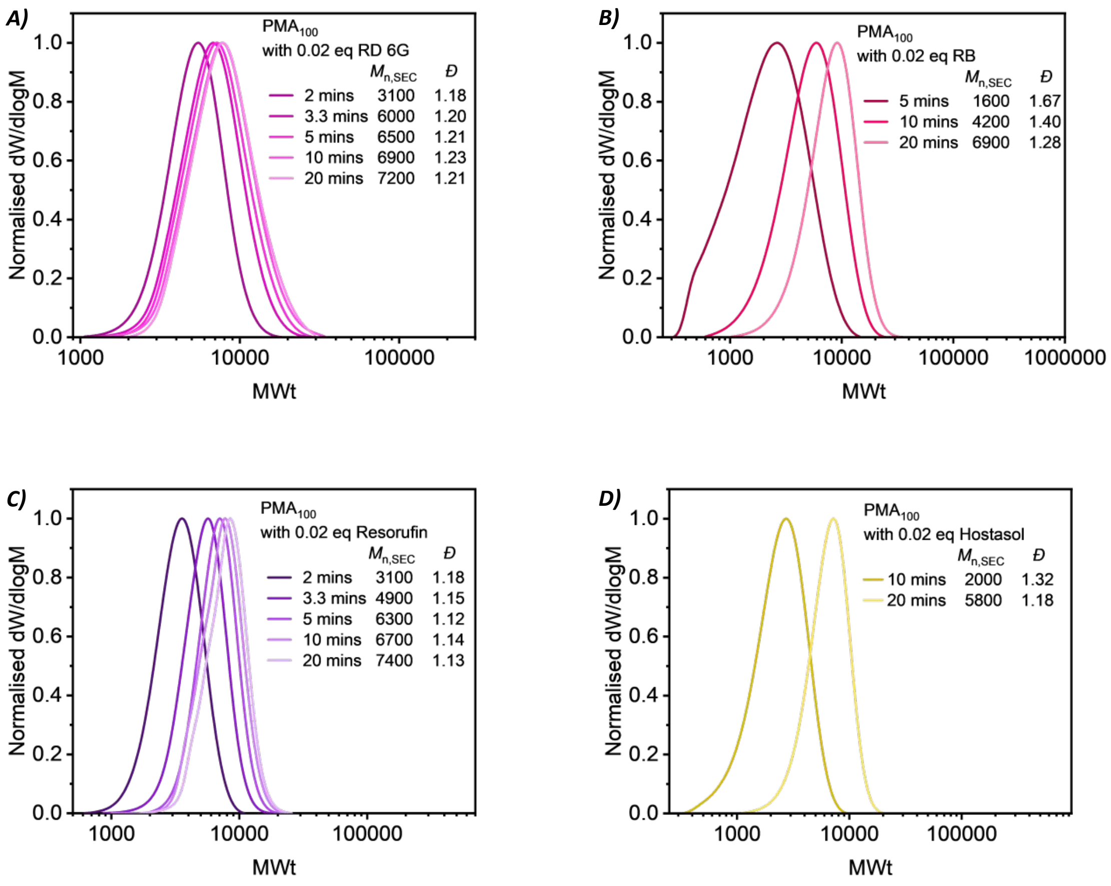

Figure S13: SEC traces showing the evolution of MWt of PMA_{100} using small amounts of A) RD 6G B) RB C) Resorufin and D) Hostasol synthesised by green light-induced Cu-RDRP in continuous flow.

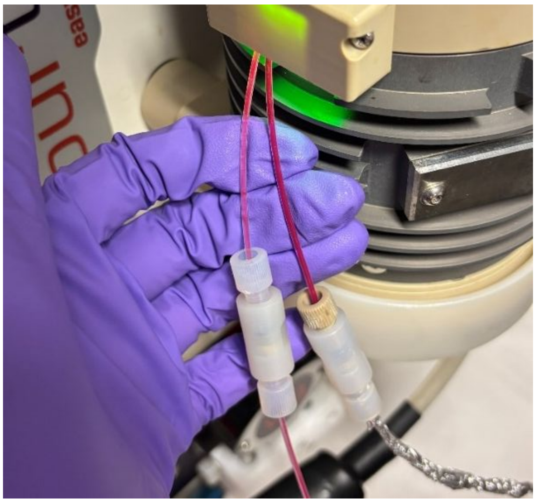

Figure S14: Image showing the colour change between the reaction mixture with resorufin entering (right tube) to exiting (left tube) from irradiation under the green light λ ~ 527 nm.

References

(1) Limer, A. J.; Rullay, A. K.; San, V.; Peinado, C.; Keely, S.; Fitzpatrick, E.; Carrington, S. D.; Brayden, D.; Haddleton, D. M. Fluorescently Tagged Star Polymers by Living Radical Polymerisation for Mucoadhesion and Bioadhesion. *React. Funct. Polym.* **2006**, *66*, 51–64. https://doi.org/10.1016/j.reactfunctpolym.2005.07.024.

(2) Ciampolini, M.; Nardi, N. Five-Coordinated High-Spin Complexes of Bivalent Cobalt, Nickel, and Copper with Tris(2-Dimethylaminoethyl)Amine. *Inorg. Chem.* **1966**, *5* (1), 41–44.

(3) Van Herck, J.; Abeysekera, I.; Buckinx, A.-L.; Cai, K.; Hooker, J.; Thakur, K.; Van de Reydt, E.; Voorter, P.-J.; Wyers, D.; Junkers, T. Operator-Independent High-Throughput Polymerization Screening Based on Automated Inline NMR and Online SEC. *Digital Discovery* **2022**, *1* (4), 519–526. https://doi.org/10.1039/d2dd00035k.

(4) Bereś, M. A.; Zhang, B.; Junkers, T.; Perrier, S. Kinetic Investigation of Photoiniferter-RAFT Polymerization in Continuous Flow Using Inline NMR Analysis. *Polym. Chem.* **2024**, *15*, 3166–3175. https://doi.org/10.1039/d4py00409d.

(5) Van Herck, J.; Junkers, T. Rapid Kinetic Screening via Transient Timesweep Experiments in Continuous Flow Reactors. *Chemistry–Methods* **2022**, *2* (1), 1–6. https://doi.org/10.1002/cmtd.202100090.

(6) Rubens, M.; Van Herck, J.; Junkers, T. Automated Polymer Synthesis Platform for Integrated Conversion Targeting Based on Inline Benchtop NMR. *ACS Macro Lett.* **2019**, *8* (11), 1437–1441. https://doi.org/10.1021/acsmacrolett.9b00767.
